# 4-Methyl-2-*n*-propyl-1*H*-benzimidazole-6-carboxylic acid

**DOI:** 10.1107/S1600536808043420

**Published:** 2009-01-08

**Authors:** Bing Xu, Ling Yong, Dan Wang, Ren Zhang, Xiang Li

**Affiliations:** a Department of Biological & Food Engineering, Changshu Institute of Technology, Changshu 215500, People’s Republic of China; b Center of Drug Discovery, China Pharmaceutical University, Nanjing 210009, People’s Republic of China; c Bioengineering Department, Xuzhou Higher Vocational College of Bioengineering, Mine West Road, Xuzhou, Xuzhou 221006, People’s Republic of China; d Research & Development, Xuzhou Nhwa Pharma Corporation, Zhongshan North Road Xuzhou, Xuzhou 221007, People’s Republic of China

## Abstract

In the title compound, C_12_H_14_N_2_O_2_, the benzene ring and imidazole ring are almost coplanar, making a dihedral angle of 2.47 (14)°. Inter­molecular O—H⋯N, N—H⋯O and C—H⋯O hydrogen bonds stabilize the crystal structure.

## Related literature

For the use of the title compound as an inter­mediate in the preparation of telmisartan, see: Ries *et al.* (1993 ▶). For the biological activity of telmisartan, see: Engeli *et al.* (2000 ▶); Goossens *et al.* (2003 ▶). For reference structural data, see: Allen *et al.* (1987 ▶). For related literature, see: Kintscher *et al.* (2004 ▶).
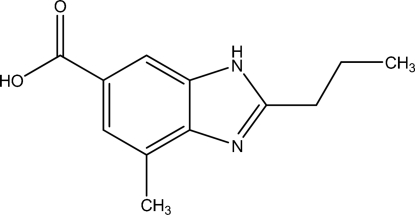


## Experimental

### 

#### Crystal data

C_12_H_14_N_2_O_2_
*M* *_r_* = 218.25
Tetragonal, 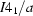

*a* = 12.0548 (17) Å
*c* = 31.899 (6) Å
*V* = 4635.5 (13) Å^3^
*Z* = 16
Mo *K*α radiation
μ = 0.09 mm^−1^
*T* = 293 (2) K
0.30 × 0.30 × 0.10 mm

#### Data collection

Enraf–Nonuis CAD-4 diffractometer
Absorption correction: ψ scan (North *et al.*, 1968 ▶) *T*_min_ = 0.975, *T*_max_ = 0.991
4426 measured reflections
2105 independent reflections
1304 reflections with *I* > 2σ(*I*)
*R*_int_ = 0.042
3 standard reflections every 200 reflections intensity decay: none

#### Refinement

*R*[*F* ^2^ > 2σ(*F* ^2^)] = 0.072
*wR*(*F* ^2^) = 0.195
*S* = 1.00
2105 reflections
133 parameters
2 restraints
H-atom parameters constrained
Δρ_max_ = 0.62 e Å^−3^
Δρ_min_ = −0.39 e Å^−3^

### 

Data collection: *CAD-4 Software* (Enraf–Nonius,1989 ▶); cell refinement: *CAD-4 Software*; data reduction: *XCAD4* (Harms & Wocadlo,1995 ▶); program(s) used to solve structure: *SHELXS97* (Sheldrick, 2008 ▶); program(s) used to refine structure: *SHELXL97* (Sheldrick, 2008 ▶); molecular graphics: *SHELXTL* (Sheldrick, 2008 ▶); software used to prepare material for publication: *SHELXTL*.

## Supplementary Material

Crystal structure: contains datablocks global, I. DOI: 10.1107/S1600536808043420/sj2567sup1.cif

Structure factors: contains datablocks I. DOI: 10.1107/S1600536808043420/sj2567Isup2.hkl

Additional supplementary materials:  crystallographic information; 3D view; checkCIF report

## Figures and Tables

**Table 1:** Hydrogen-bond geometry (Å, °)

*D*—H⋯*A*	*D*—H	H⋯*A*	*D*⋯*A*	*D*—H⋯*A*
C3—H3*B*⋯O2^i^	0.97	2.56	3.399 (4)	144
C11—H11*A*⋯O2^ii^	0.96	2.66	3.587 (4)	163
O2—H2*C*⋯N1^iii^	0.82	1.80	2.562 (3)	153
N2—H2*D*⋯O1^iv^	0.86	1.83	2.673 (3)	165

Symmetry codes: (i) 
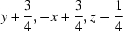
; (ii) 
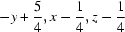
; (iii) 
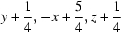
; (iv) 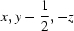.

**Fig. 1.**
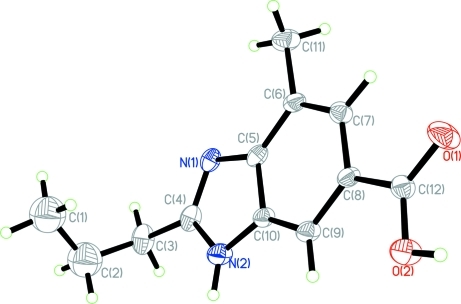
The molecular structure of the title molecule, with the atom-numbering scheme. Displacement ellipsoids are drawn at the 30% probability level.

**Fig. 2.**
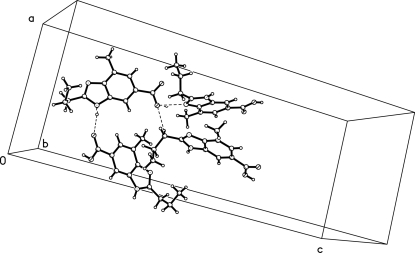
Crystal packing of the title compound with hydrogen bonds drawn as dashed lines.

## supplementary crystallographic information

### Comment

The title compound (I), Fig. 1, 4-methyl-2-n-propyl-1*H*-benzimidazole-6-carboxylic acid is an important intermediate in the preparation of the angiotensin II receptor blocker telmisartan (Ries *et al.*,1993). Telmisartan is used as a therapeutic tool for metabolic problems, including visceral obesity (Engeli *et al.*, 2000; Goossens *et al.*, 2003). Bond lengths in the compound are within normal ranges (Allen *et al.*, 1987) and the benzene and imidazole rings are coplanar with a dihedral angle of 2.47 (14)° between them.

In the crystal structure, intermolecular O—H···N, N—H···O and C—H···O hydrogen bonds, Table 1, link the molecules, Fig. 2, and stabilise the crystal packing.

### Experimental

The title compound was prepared from methyl 4-(butyrylamino)-5-methyl -3-aminobenzoate (13 g 52 mmol) in xylene (60 mL) and hydrochloric acid (130 mL). The mixture was refluxed for 3 h at 423 K, concentrated under reduced pressure then 80mL of methanol and 110 mL 15% sodium hydroxide were added. This solution was further , refluxed for 5 h at 383 K. The pH was adjusted to below 7 and the product filtered. Recrystallization of product from a mixture of ethanol/water (5:1) afforded the white powder. Crystals were obtained by dissolving the product (1.0 g) in acetone (10 ml) and evaporating slowly at room temperature for about 30 d.

### Refinement

H All atoms were positioned geometrically, with N—H = 0.86 Å (for NH) and C—H = 0.93, 0.97 and 0.96 Å for aromatic, methine and methyl H, and constrained to ride on their parent atoms, with *U*_iso_(H) = *xU*_eq_(C,*N*), where *x* = 1.5 for methyl H, and *x* = 1.2 for all other H atoms.

### Crystal data

**Table:**

C_12_H_14_N_2_O_2_	*D*_x_ = 1.251 Mg m^−^^3^
*M**_r_* = 218.25	Mo *K*α radiation, λ = 0.71073 Å
Tetragonal, *I*4_1_/*a*	Cell parameters from 25 reflections
Hall symbol: -I 4ad	θ = 10–13°
*a* = 12.0548 (17) Å	µ = 0.09 mm^−^^1^
*c* = 31.899 (6) Å	*T* = 293 K
*V* = 4635.5 (13) Å^3^	Block, colorless
*Z* = 16	0.30 × 0.30 × 0.10 mm
*F*(000) = 1856	

### Data collection

**Table:**

Enraf–Nonuis CAD-4 diffractometer	1304 reflections with *I* > 2σ(*I*)
Radiation source: fine-focus sealed tube	*R*_int_ = 0.042
graphite	θ_max_ = 25.2°, θ_min_ = 1.8°
ω/2θ scans	*h* = −9→10
Absorption correction: ψ scan (North *et al.*, 1968)	*k* = 0→14
*T*_min_ = 0.975, *T*_max_ = 0.991	*l* = 0→38
4426 measured reflections	3 standard reflections every 200 reflections
2105 independent reflections	intensity decay: none

### Refinement

**Table:**

Refinement on *F*^2^	Secondary atom site location: difference Fourier map
Least-squares matrix: full	Hydrogen site location: inferred from neighbouring sites
*R*[*F*^2^ > 2σ(*F*^2^)] = 0.072	H-atom parameters constrained
*wR*(*F*^2^) = 0.195	*w* = 1/[σ^2^(*F*_o_^2^) + (0.1*P*)^2^ + 5*P*] where *P* = (*F*_o_^2^ + 2*F*_c_^2^)/3
*S* = 1.00	(Δ/σ)_max_ < 0.001
2105 reflections	Δρ_max_ = 0.62 e Å^−^^3^
133 parameters	Δρ_min_ = −0.39 e Å^−^^3^
2 restraints	Extinction correction: *SHELXL97* (Sheldrick, 2008)
Primary atom site location: structure-invariant direct methods	Extinction coefficient: 0.039 (3)

### Special details

**Table:**

Geometry. All e.s.d.'s (except the e.s.d. in the dihedral angle between two l.s. planes) are estimated using the full covariance matrix. The cell e.s.d.'s are taken into account individually in the estimation of e.s.d.'s in distances, angles and torsion angles; correlations between e.s.d.'s in cell parameters are only used when they are defined by crystal symmetry. An approximate (isotropic) treatment of cell e.s.d.'s is used for estimating e.s.d.'s involving l.s. planes.
Refinement. Refinement of *F*^2^ against ALL reflections. The weighted *R*-factor *wR* and goodness of fit *S* are based on *F*^2^, conventional *R*-factors *R* are based on *F*, with *F* set to zero for negative *F*^2^. The threshold expression of *F*^2^ > σ(*F*^2^) is used only for calculating *R*-factors(gt) *etc*. and is not relevant to the choice of reflections for refinement. *R*-factors based on *F*^2^ are statistically about twice as large as those based on *F*, and *R*- factors based on ALL data will be even larger.

### Fractional atomic coordinates and isotropic or equivalent isotropic displacement parameters (Å^2^)

**Table:**

	*x*	*y*	*z*	*U*_iso_*/*U*_eq_	
O1	0.7815 (2)	0.4947 (2)	0.07388 (7)	0.0591 (8)	
O2	0.7256 (2)	0.32125 (18)	0.07006 (6)	0.0471 (7)	
H2C	0.7077	0.3341	0.0944	0.071*	
N1	0.8807 (2)	0.3584 (2)	−0.11544 (7)	0.0376 (7)	
N2	0.8170 (2)	0.2123 (2)	−0.08282 (7)	0.0420 (7)	
H2D	0.7940	0.1458	−0.0785	0.050*	
C1	0.6545 (5)	0.2486 (5)	−0.18175 (17)	0.104	
H1B	0.5891	0.2171	−0.1941	0.155*	
H1C	0.6843	0.3042	−0.2001	0.155*	
H1D	0.6358	0.2818	−0.1553	0.155*	
C2	0.7407 (4)	0.1577 (5)	−0.17491 (18)	0.100	
H2A	0.7097	0.1043	−0.1554	0.120*	
H2B	0.7513	0.1195	−0.2014	0.120*	
C3	0.8501 (3)	0.1904 (3)	−0.15925 (10)	0.0561 (10)	
H3A	0.8860	0.2355	−0.1805	0.067*	
H3B	0.8945	0.1239	−0.1557	0.067*	
C4	0.8509 (3)	0.2525 (3)	−0.11926 (9)	0.0402 (8)	
C5	0.8656 (2)	0.3874 (3)	−0.07355 (9)	0.0324 (7)	
C6	0.8820 (2)	0.4885 (2)	−0.05283 (9)	0.0341 (7)	
C7	0.8539 (2)	0.4896 (3)	−0.01107 (9)	0.0351 (7)	
H7A	0.8653	0.5543	0.0042	0.042*	
C8	0.8085 (2)	0.3966 (2)	0.00960 (9)	0.0315 (7)	
C9	0.7938 (3)	0.2969 (2)	−0.01128 (9)	0.0353 (8)	
H9A	0.7645	0.2350	0.0021	0.042*	
C10	0.8246 (3)	0.2941 (2)	−0.05289 (9)	0.0325 (7)	
C11	0.9269 (3)	0.5876 (3)	−0.07572 (11)	0.0523 (10)	
H11A	0.9410	0.5681	−0.1044	0.078*	
H11B	0.9947	0.6112	−0.0627	0.078*	
H11C	0.8737	0.6467	−0.0747	0.078*	
C12	0.7704 (3)	0.4069 (2)	0.05433 (9)	0.0352 (7)	

### Atomic displacement parameters (Å^2^)

**Table:**

	*U*^11^	*U*^22^	*U*^33^	*U*^12^	*U*^13^	*U*^23^
O1	0.097 (2)	0.0411 (14)	0.0391 (14)	−0.0157 (14)	0.0171 (13)	−0.0130 (11)
O2	0.0713 (17)	0.0394 (13)	0.0306 (12)	−0.0073 (12)	0.0238 (11)	−0.0056 (10)
N1	0.0401 (16)	0.0475 (17)	0.0250 (13)	0.0030 (13)	0.0022 (11)	0.0055 (12)
N2	0.0599 (19)	0.0366 (15)	0.0295 (13)	−0.0022 (13)	0.0098 (13)	0.0006 (12)
C1	0.104	0.104	0.104	0.000	0.000	0.000
C2	0.100	0.100	0.100	0.000	0.000	0.000
C3	0.062 (2)	0.079 (3)	0.0275 (18)	0.006 (2)	0.0032 (16)	−0.0106 (17)
C4	0.0392 (19)	0.056 (2)	0.0250 (16)	0.0079 (16)	0.0066 (14)	0.0000 (15)
C5	0.0309 (16)	0.0392 (18)	0.0271 (15)	0.0063 (13)	0.0052 (13)	0.0077 (13)
C6	0.0356 (17)	0.0303 (17)	0.0365 (17)	−0.0015 (13)	0.0005 (14)	0.0093 (13)
C7	0.0379 (18)	0.0330 (17)	0.0345 (16)	−0.0008 (13)	0.0032 (13)	−0.0027 (13)
C8	0.0410 (18)	0.0273 (16)	0.0261 (15)	0.0038 (13)	0.0033 (13)	0.0040 (12)
C9	0.0472 (19)	0.0318 (17)	0.0269 (16)	−0.0020 (14)	0.0091 (13)	0.0032 (13)
C10	0.0454 (19)	0.0293 (16)	0.0229 (14)	0.0015 (13)	0.0046 (13)	0.0015 (12)
C11	0.064 (2)	0.041 (2)	0.052 (2)	−0.0089 (17)	0.0104 (18)	0.0146 (17)
C12	0.0430 (19)	0.0321 (17)	0.0305 (16)	0.0009 (14)	0.0040 (14)	−0.0042 (14)

### Geometric parameters (Å, °)

**Table:**

O1—C12	1.237 (4)	C3—H3A	0.9700
O2—C12	1.268 (3)	C3—H3B	0.9700
O2—H2C	0.8200	C5—C10	1.394 (4)
N1—C4	1.332 (4)	C5—C6	1.400 (4)
N1—C5	1.393 (4)	C6—C7	1.374 (4)
N2—C4	1.324 (4)	C6—C11	1.501 (4)
N2—C10	1.376 (4)	C7—C8	1.411 (4)
N2—H2D	0.8600	C7—H7A	0.9300
C1—C2	1.526 (6)	C8—C9	1.385 (4)
C1—H1B	0.9600	C8—C12	1.504 (4)
C1—H1C	0.9600	C9—C10	1.379 (4)
C1—H1D	0.9600	C9—H9A	0.9300
C2—C3	1.465 (6)	C11—H11A	0.9600
C2—H2A	0.9700	C11—H11B	0.9600
C2—H2B	0.9700	C11—H11C	0.9600
C3—C4	1.479 (4)		
			
C12—O2—H2C	109.5	N1—C5—C6	130.7 (3)
C4—N1—C5	107.0 (2)	C10—C5—C6	121.9 (3)
C4—N2—C10	109.1 (3)	C7—C6—C5	115.5 (3)
C4—N2—H2D	125.5	C7—C6—C11	123.5 (3)
C10—N2—H2D	125.5	C5—C6—C11	120.9 (3)
C2—C1—H1B	109.5	C6—C7—C8	122.7 (3)
C2—C1—H1C	109.5	C6—C7—H7A	118.6
H1B—C1—H1C	109.5	C8—C7—H7A	118.6
C2—C1—H1D	109.5	C9—C8—C7	120.9 (3)
H1B—C1—H1D	109.5	C9—C8—C12	119.2 (3)
H1C—C1—H1D	109.5	C7—C8—C12	119.8 (3)
C3—C2—C1	118.0 (5)	C10—C9—C8	116.7 (3)
C3—C2—H2A	107.8	C10—C9—H9A	121.6
C1—C2—H2A	107.8	C8—C9—H9A	121.6
C3—C2—H2B	107.8	N2—C10—C9	131.9 (3)
C1—C2—H2B	107.8	N2—C10—C5	105.9 (2)
H2A—C2—H2B	107.2	C9—C10—C5	122.1 (3)
C2—C3—C4	115.8 (4)	C6—C11—H11A	109.5
C2—C3—H3A	108.3	C6—C11—H11B	109.5
C4—C3—H3A	108.3	H11A—C11—H11B	109.5
C2—C3—H3B	108.3	C6—C11—H11C	109.5
C4—C3—H3B	108.3	H11A—C11—H11C	109.5
H3A—C3—H3B	107.4	H11B—C11—H11C	109.5
N2—C4—N1	110.7 (3)	O1—C12—O2	122.9 (3)
N2—C4—C3	124.8 (3)	O1—C12—C8	121.1 (3)
N1—C4—C3	124.5 (3)	O2—C12—C8	116.0 (3)
N1—C5—C10	107.3 (3)		
			
C1—C2—C3—C4	56.3 (6)	C6—C7—C8—C12	−174.6 (3)
C10—N2—C4—N1	−0.2 (4)	C7—C8—C9—C10	−0.6 (5)
C10—N2—C4—C3	−177.3 (3)	C12—C8—C9—C10	176.5 (3)
C5—N1—C4—N2	0.5 (4)	C4—N2—C10—C9	175.5 (3)
C5—N1—C4—C3	177.5 (3)	C4—N2—C10—C5	−0.1 (4)
C2—C3—C4—N2	63.9 (5)	C8—C9—C10—N2	−176.8 (3)
C2—C3—C4—N1	−112.7 (4)	C8—C9—C10—C5	−1.8 (5)
C4—N1—C5—C10	−0.5 (3)	N1—C5—C10—N2	0.4 (3)
C4—N1—C5—C6	−178.6 (3)	C6—C5—C10—N2	178.6 (3)
N1—C5—C6—C7	177.2 (3)	N1—C5—C10—C9	−175.7 (3)
C10—C5—C6—C7	−0.6 (4)	C6—C5—C10—C9	2.5 (5)
N1—C5—C6—C11	−2.5 (5)	C9—C8—C12—O1	−179.5 (3)
C10—C5—C6—C11	179.7 (3)	C7—C8—C12—O1	−2.4 (5)
C5—C6—C7—C8	−1.8 (4)	C9—C8—C12—O2	−0.4 (4)
C11—C6—C7—C8	177.9 (3)	C7—C8—C12—O2	176.7 (3)
C6—C7—C8—C9	2.4 (5)		

### Hydrogen-bond geometry (Å, °)

**Table:**

*D*—H···*A*	*D*—H	H···*A*	*D*···*A*	*D*—H···*A*
C3—H3B···O2^i^	0.97	2.56	3.399 (4)	144
C11—H11A···O2^ii^	0.96	2.66	3.587 (4)	163
O2—H2C···N1^iii^	0.82	1.80	2.562 (3)	153
N2—H2D···O1^iv^	0.86	1.83	2.673 (3)	165

Symmetry codes: (i) *y*+3/4, −*x*+3/4, *z*−1/4; (ii) −*y*+5/4, *x*−1/4, *z*−1/4; (iii) *y*+1/4, −*x*+5/4, *z*+1/4; (iv) *x*, *y*−1/2, −*z*.
